# Integrating multidisciplinary team teaching with case-based learning in urology internship education

**DOI:** 10.1186/s12909-026-08730-w

**Published:** 2026-02-06

**Authors:** Shiying Tang, Shaohui Deng, Yu Tian, Min Qiu, Shudong Zhang

**Affiliations:** Department of Urology, Peking University Third Hospital, Beijing, 100191 China

**Keywords:** Multidisciplinary team teaching model, Clinical medicine program, Interns, CBL teaching method, Urology teaching

## Abstract

**Objective:**

To evaluate the feasibility and initial efficacy of a multidisciplinary team (MDT)-enhanced case-based learning (CBL) approach within urology clinical internship training.

**Methods:**

A pilot, cluster-randomized controlled study was conducted at Peking University Third Hospital from January 2022 to September 2023. Eighty-four medical interns in clinical medicine were allocated by pre-formed training groups: 32 to the experimental (MDT-CBL: multidisciplinary team-enhanced case-based learning) group and 52 to the control (traditional teaching) group. The MDT-CBL model integrated urologists, radiologists, and ultrasonographers in teaching structured around real clinical cases. Outcomes included a 20-item written test on urological knowledge and a questionnaire assessing teaching method preference before and after the intervention.

**Results:**

Interns in the MDT-CBL group achieved a higher mean score on the knowledge test (6.2 ± 2.5) compared to the control group (5.8 ± 3.4), though this difference did not reach statistical significance (*p* = 0.52). Subjectively, the intervention was highly accepted: 65.6% (21/32) of interns in the experimental group preferred the MDT-CBL model post-intervention, with 17 interns switching their preference from the traditional method. This indicates a strong positive shift in learner engagement.

**Conclusion:**

The integration of an MDT framework with CBL in urology internship training presents a well-accepted and engaging educational model. While this pilot study did not show a statistically significant advantage over traditional teaching in a written test of foundational knowledge, it was associated with significantly higher learner satisfaction and preference. These findings suggest the model’s primary value may lie in enriching the learning experience and fostering early interdisciplinary collaboration.

## Introduction

In the context of ongoing healthcare reform in China, particularly following the “Healthy China 2030” blueprint [1], there is an escalating emphasis on cultivating clinicians with robust collaborative practice and systems-thinking abilities. This evolution places heightened demands on medical education to adapt its training models. Medical colleges and universities, as the primary bases for cultivating medical talent, play a crucial role in equipping students with clinical expertise. In the Chinese context, undergraduate medical students (typically in a 5-year program) enter their final year of study, which is dedicated to clinical internships across multiple disciplines (e.g., Internal Medicine, Surgery, Pediatrics, Urology). The educational intervention in this study was implemented during the urology internship rotation within this final year. Upon graduation, students enter the national Post-Graduate Residency Training system. Urology is a clinical specialty requiring multidisciplinary collaboration and multi-system diagnosis and treatment, involving disciplines such as internal medicine, surgery, radiology, ultrasonography, and nuclear medicine. Consequently, the teaching of urological imaging and ultrasonography holds significant importance during urology internships.

However, in actual clinical practice, urology specialists often lack extensive diagnostic and clinical experience specifically in urological imaging and ultrasound. When teaching these topics themselves, they can only cover aspects within their own specialty, resulting in less rich content and limited sharing of clinical experience compared to dedicated radiologists or ultrasonographers. To mitigate these shortcomings, the “multidisciplinary team (MDT)” teaching model has gradually been adopted in medical education at renowned institutions both domestically and internationally [2]. On the other hand, the traditional lecture-based learning (LBL) method primarily focuses on systematic theoretical instruction by the teacher, neglecting interactive components and the application of knowledge to real clinical cases. This approach is deficient in cultivating students’ comprehensive clinical abilities and practical problem-solving skills for clinical diseases [3, 4]. The case-based learning (CBL) method, a student-centered, discussion-based approach guided by clinical cases, has been widely adopted and practiced by most medical schools worldwide [5]. After exposure to traditional teaching content, students encounter real clinical cases. They independently identify patient problems using learned methods like history taking and physical examination, apply prior disciplinary knowledge to analyze the clinical case, and propose diagnostic and therapeutic plans.

This study aims to investigate the role and feasibility of the CBL teaching method based on a MDT teaching model in the urology clinical internship teaching for clinical medicine program students.

## Methods

### Research subjects

**Fig. 1:**
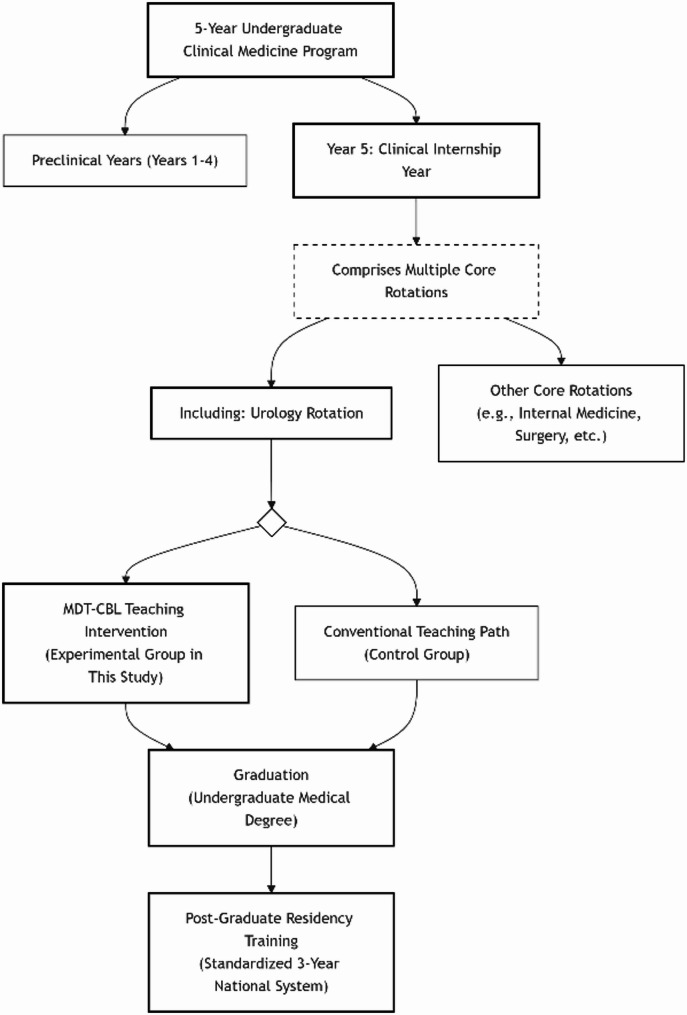
Educational pathway of study participants and placement of the intervention in China

Two groups totaling 84 clinical medicine program students undertaking internships at our hospital from January 2022 to September 2023 were selected for urology clinical teaching. Prior to the intervention, all students had completed core preclinical courses (including anatomy, physiology, pathophysiology, etc.), but had not received specialized training in urology, urological imaging or ultrasound interpretation. The standard Chinese medical education pathway and the precise placement of this educational intervention within it are illustrated in Fig. 1.

### Randomization procedure

This study employed a cluster-randomized design. A total of 3 pre-formed intern training groups (clusters) were used for randomization. The allocation of students into these 3 different training groups was performed by the hospital’s Education Office at the start of the internship rotation using a computer-generated random number sequence. Two clusters (total *n* = 52 interns) were then randomly selected as the control group, and one cluster (*n* = 32 interns) was selected as the experimental group. This approach was taken to minimize contamination between teaching methods within the same clinical rotation period. The sample size was determined by the number of interns available during the study period, representing a convenience sample for this initial pilot study.

### Research methods

Instructors developed the teaching plan through faculty training and collective lesson planning sessions. A pre-teaching questionnaire was designed to gauge student preferences (including questions like: “Before your urology internship, which teaching method do you prefer? ① Relevant content taught by specialist B-ultrasound and Radiology teachers. ② Relevant B-ultrasound and imaging content taught by a urologist.“). The clinical cases used were selected for their relevance to common urological conditions and their suitability for illustrating key teaching points across urology, radiology, and ultrasonography. All cases were reviewed and standardized by the MDT teaching team to ensure consistency and appropriateness for the intern’s learning level. Teaching aids were prepared, and post-teaching assessment content was designed. Students were required to prepare for the learning content beforehand using textbooks and online resources for preview and self-study. The course lasts approximately 4 h.

### MDT-CBL group (experimental group)

The MDT teaching team was specifically convened for this educational intervention. It consisted of: (1) a lead urology attending physician who coordinated the case discussion and integrated clinical management points; (2) a consultant radiologist specializing in genitourinary imaging, responsible for teaching and interpreting CT or MRI findings relevant to the cases; (3) a consultant ultrasonographer specializing in urological ultrasound, responsible for teaching the principles, techniques, and interpretation of renal, bladder, and prostate ultrasounds. All MDT members participated in joint lesson planning to ensure content alignment and were present during the dedicated “small-group sessions” for the experimental group.

Instructors organized students to conduct history taking and physical examinations on actual clinical patients, followed by a structured group discussion to refine the diagnosis and its basis, differential diagnoses, and the next steps in management. During discussion and reporting, instructors provided commentary and guidance, adjusted and optimized teaching content based on the discussion, and answered individual student questions, helping each student understand their strengths and weaknesses. Finally, the instructor consolidated the learning and summarized key points. During the unified teaching phase, content involving radiological imaging and ultrasonography was taught and clarified by specialist physicians (radiologist, nuclear medicine physician) using powerpoint presentations.

### Traditional teaching method group (control group)

The instructor first delivered a unified lecture covering the session’s content. During this phase, content involving radiological imaging and ultrasonography was taught and clarified by the urologist. Subsequently, students were organized to conduct history taking and physical examinations on actual clinical patients, followed by a structured group discussion to refine the diagnosis and its basis, differential diagnoses, and the next steps in management. Instructors provided commentary, guidance, and answered questions during the discussion.

### Post-Teaching feedback

Feedback was collected via a questionnaire and a written examination. The post-teaching questionnaire mirrored the pre-teaching one (e.g., “After your urology internship, which teaching method do you prefer? ①…②…”). The written examination consisted of 10 questions on upper urinary tract diseases (5 points) and 10 questions on lower urinary tract diseases (5 points), totaling 10 points. The 20-item written examination was developed collaboratively by the full MDT teaching team (including urologists, radiologists, and ultrasonographers) to ensure it assessed the integrated learning objectives. Crucially, all test items were mapped to the core urological knowledge and clinical reasoning skills that are standard components of the internship curriculum. This ensured that although the control group received the relevant imaging content from a urologist rather than imaging specialists, they were still taught and were expected to learn the foundational concepts necessary to answer all examination questions. Only one post-intervention test was administered to each group immediately following the teaching session.

### Effect evaluation

Pre-teaching questionnaires were administered to both groups to understand their initial inclination towards the teaching models. Post-teaching questionnaires and the written examination were administered to both groups after the internship. This assessed the experimental group’s preference and subjective evaluation of the new model after experiencing it. The written examination objectively evaluated the learning outcomes of both groups. The survey results and test scores were subjected to statistical analysis.

### Statistical methods

SPSS 22.0 software was used for statistical analysis. Categorical data were expressed as n (%) and analyzed using the χ² test. Continuous data (examination scores) were expressed as mean ± standard deviation (x̄ ± s). Due to the relatively small sample size and non-normal distribution of score data as assessed by the Shapiro-Wilk test, between-group comparisons of scores were performed using the non-parametric Mann-Whitney U test. To quantify the magnitude of between-group differences, effect sizes were calculated as Cohen’s d based on means and pooled standard deviations. A P-value < 0.05 was considered statistically significant.

## Results

As summarized in Table 1, interns in the MDT-CBL group achieved numerically higher mean scores on the theoretical knowledge test compared to those in the traditional teaching group, both for upper urinary tract diseases (2.5 vs. 2.2), lower urinary tract diseases (4.0 vs. 3.7), and in the total score (6.2 vs. 5.8). However, none of these differences reached statistical significance (all *p* > 0.05). Regarding the acceptance of the new teaching model, among the interns in the experimental group exposed to the new model, 21 interns (65.6%) preferred it after the internship. Notably, 17 of these interns (53.1% of the experimental group) had initially indicated a preference for the traditional model before the internship, demonstrating a clear shift toward the new model (see Table 2). A McNemar test revealed a statistically significant change in teaching model preference following the intervention (χ² = 15.059, *p* < 0.001).

**Table 1:** Comparison of teaching performance between experimental and control groups

Group	*n*	Upper Urinary Tract Score (x̄ ± s)	Lower Urinary Tract Score (x̄ ± s)	Total Score (x̄ ± s)
Experimental	32	2.5 ± 1.1	4 ± 0.8	6.2 ± 2.5
Control	52	2.2 ± 1.5	3.7 ± 0.8	5.8 ± 3.4
*P*-value (95%CI)		0.94(-0.41, 1.01)	0.17(-0.13, 0.73)	0.52(-0.86, 1.66)
Effect Size (Cohen’s d)		0.22	0.38	0.14

**Table 2:** Evaluation of teaching model preference by experimental group interns before and after teaching

Preference Before Teaching	*n*	Preference After Teaching	*n*	*P* value
Preferred Team Model	4	Preferred Team Model	21	
Preferred Traditional Model	28	Preferred Traditional Model	11	< 0.001

In summary, while the MDT-CBL approach did not lead to a statistically significant increase in post-test scores within this pilot sample, it was associated with a strong and statistically significant shift in learner preference. A majority of participants who experienced the integrated model favored it over the traditional approach after the internship.

## Discussion

Urology is a clinical specialty demanding multidisciplinary collaboration and multi-system diagnosis and treatment, involving disciplines such as radiology, ultrasonography, radiation oncology, and medical oncology. During urology internships, students must not only learn the fundamentals of urological diseases but also acquire knowledge in ultrasonography, imaging, and integrate knowledge across disciplines to enhance their diagnostic and therapeutic capabilities for urological conditions. Traditional CBL is a discussion-based teaching method centered on real clinical cases, aiming to cultivate students’ ability to apply learned knowledge to solve practical clinical problems [6]. The CBL teaching method based on a MDT teaching model is a novel medical education approach designed to improve teaching quality. Its main characteristic involves students, who have already acquired foundational medical knowledge, discussing real clinical cases based on learned urology specialty knowledge to identify difficulties and weaknesses in their understanding. Subsequently, a qualified urology specialist instructor leads a team of specialist instructors from multiple departments (e.g., radiology, ultrasound) to jointly teach a specific disease. Its core emphasizes close interdisciplinary collaboration and knowledge complementarity, fostering a complete clinical reasoning process in students – from symptoms and signs, through auxiliary investigations, to diagnosis and treatment. The development of analytical depth through MDT-guided dialogue holds particular significance for clinical interns. Contemporary studies on AI-assisted clinical reasoning identify “interpretive depth” as a critical benchmark [7]. For interns, as for current AI systems, achieving this depth remains a central challenge. While AI outputs can be superficial or fragmented, the MDT-CBL framework is deliberately structured to mentor interns in this essential competency. Through direct observation of and participation in real-time case discussions with urologists, radiologists, and ultrasonographers, interns practice synthesizing clinical history, imaging details, and laboratory data into a logically coherent diagnostic story. This educational exposure actively demonstrates and instills the kind of integrative, reflective reasoning that underpins expert clinical judgment, offering a distinct pedagogical strength compared to both conventional didactic teaching and existing AI-based decision supports.

The results of this study indicate that after experiencing the MDT-CBL method, objective evaluation via the urology written examination showed that the experimental group scored higher than the control group in theoretical knowledge of urinary system diseases. However, no statistically significant difference was found. The lack of statistical significance in test scores, despite positive trends, invites careful reflection. The 20-item written test, while covering core curriculum knowledge, may not be a sufficiently sensitive instrument. It underscores that measurable gains in foundational knowledge from a short-term educational intervention may be modest and difficult to detect with a small sample and a general written test, which may not fully reflect comprehensive knowledge mastery.This may also be related to the interns’ stage of learning where their grasp of urology knowledge is not yet deep. Nevertheless, the numerical improvement in scores suggests a trend towards better knowledge grasp among students exposed to the new model. Simultaneously, CBL integrates basic theoretical knowledge into specific clinical cases, helping students consolidate learned multidisciplinary clinical knowledge and tightly linking prior knowledge review with clinical practice. This approach encourages students to proactively gather and consult materials before class, leading to more efficient use of study time and enhanced self-directed learning abilities [4]. The more pronounced effect was seen in learner satisfaction and preference. Specifically, 21/32 (65.6%) students preferred the new teaching method, while the proportion still favoring the traditional model decreased by approximately half, collectively suggesting a strong subjective preference for the new approach. This suggests that the primary value of the MDT-CBL model in this pilot phase may lie in its process benefits: enhancing engagement, exposing students to authentic interdisciplinary dialogue, and modeling collaborative care. Future research should investigate whether these process benefits translate into superior clinical competency over longer periods.

The clinical diagnosis and treatment of urological diseases rely not only on routine history taking and physical examination but also heavily on auxiliary diagnostics like laboratory tests and imaging. Therefore, explaining auxiliary investigations is a crucial part of urology teaching. However, in practice, this is often done by urologists. Compared to teaching by dedicated ultrasonographers or radiologists, explanations by urologists may sometimes be less thorough or less closely linked to the nuances of image interpretation in clinical decision-making. Long-term teaching observations suggest that while the traditional model helps students memorize textbook knowledge, the lack of specialist input can weaken the connection to clinical practice, leading to knowledge attrition over time. Multidisciplinary teamwork is essential for the advancement of medicine and may become a necessity in the reform of specialized clinical medical education [8]. Consequently, we implemented the “MDT Teaching” model, selecting experienced instructors from specialties like Ultrasonography and Radiology to form a teaching team with urology specialists, enabling comprehensive teaching of urological diseases.

The traditional CBL teaching method, originally proposed by Harvard University, is a discussion-based approach centered on real cases. It typically starts with the instructor lecturing on target specialty knowledge, consistent with traditional teaching [9]. Students then directly engage with real patients, applying learned knowledge to clinical practice by formulating specific diagnostic and therapeutic plans. This process consolidates knowledge and enhances analytical and problem-solving skills. Relevant studies have proven that CBL helps stimulate students’ clinical learning interest, cultivates independent thinking regarding clinical problems, and can significantly improve clinical teaching effectiveness [10]. Despite its evident advantages and widespread adoption, traditional CBL also has limitations. A prominent issue with the conventional specialty lecture format is its reliance on experienced urologists based primarily on textbooks. This often leaves them lacking the broad clinical diagnostic and therapeutic expertise required for integrated case-based learning, particularly in supporting non-core specialties such as radiology, ultrasonography, and nuclear medicine. Consequently, they may struggle to emphasize key teaching points or draw upon accumulated practical clinical experience in these areas during teaching. To avoid this, we modified traditional CBL into the following three modules: (1) preparatory self-study: Students, having already learned relevant basic medical sciences (epidemiology, anatomy, pathophysiology) and attended urology lectures, engage in targeted learning before specific urology disease teaching. Using textbooks and clinical cases as a guide, they perform literature searches, reviews, and accumulate professional knowledge. This phase enhances student initiative, provides disease-centered problem-oriented learning, and effectively cultivates abilities in independent thinking, question posing, analysis, and problem-solving [11]. (2) traditional CBL application: Students directly interact with patients for clinical case management. During case discussions, group members collaboratively analyze and solve the case with input and guidance from the instructor. Past research shows that clinical teaching quality is significantly related to instructor teaching behaviors [12]. This approach fosters problem identification and solving skills, promotes interaction between teacher-student and student-student, and cultivates teamwork spirit, enhancing both student interest and instructor motivation [13]. (3) consolidation by specialist team: Instructors, collaborating with specialists like radiologists and ultrasonographers, deliver “small-group sessions” to systematically review and summarize key points after students have encountered the actual clinical cases. Having engaged with real patients, students can better identify their own knowledge gaps and difficulties. These “small-group sessions” provide focused learning and supplementation, improving comprehensive mastery of the subject matter. The MDT-CBL method uses urological disease case analysis as an entry point, encouraging students to review foundational clinical knowledge, guiding them through structured inquiry and critical thinking, and fostering creative practical thinking skills. The concluding summaries by the MDT after CBL discussions, combined with the unified review lectures, reinforce learning content, clarify key points, and cultivate holistic clinical reasoning, ultimately helping students form a complete clinical thought process for urogenital malignancies.

The evolution of urological education is increasingly shaped by the integration of technological innovations, particularly through the adoption of surgical simulators, augmented reality (AR), and AI-driven platforms. Recent evidence indicates that AR enhances spatial awareness, procedural accuracy, and interdisciplinary collaboration in surgical training, closely aligning with the goals of multidisciplinary, case-based learning [14, 15]. While AR and AI-driven simulations typically prioritize the development of technical skills, our MDT-CBL model places a stronger emphasis on fostering collaborative clinical reasoning, effective communication, and integrative decision-making in real-world, multidisciplinary team contexts. Together, these methods form complementary strands of modern urological education, wherein AI can generate structured cases and provide real-time decision support within the MDT-CBL framework, while AR visualizes anatomy and surgical pathways during multidisciplinary discussions. This synergy between technology-enhanced simulation and team-based case learning fosters the development of clinically competent, context-aware future urologists skilled in both technical proficiency and collaborative patient care.

This study has also some limitations: First, the modest sample size and unequal group allocation reduced statistical power, likely explaining the lack of significance in exam scores despite numerical improvements; Second, the brief, single-session nature of the intervention (approximately 4 h) may limit its ability to produce substantial and measurable changes in knowledge outcomes among interns with varied prior clinical exposure; Third, the use of a 20-question written test, while covering essential curriculum components, may not capture the full breadth of knowledge acquisition, limiting comprehensive assessment of the intervention’s impact on practical skill development; Fourth, the assessment of learner preference relied on subjective questionnaires. While the observed shift was significant, the positive ratings could be partly influenced by a short-term novelty effect associated with the new teaching format, which is a common limitation in evaluating educational innovations. Future studies would benefit from more comprehensive and longitudinal assessments, such as objective structured clinical examinations to evaluate practical skills, validated instruments to measure clinical reasoning, and delayed post-tests (e.g., at 3 or 6 months) to assess knowledge retention and transfer to clinical practice.

In conclusion, the CBL teaching method based on a MDT teaching model represents a promising and well-accepted alternative to conventional teaching methods. While this pilot study did not demonstrate a statistically significant advantage of the MDT-CBL model over traditional teaching in a written test of foundational knowledge, it was associated with significantly higher learner satisfaction and preference. This suggests the model’s primary value in this context may lie in enhancing the learning experience and fostering early exposure to interdisciplinary collaboration, meriting further exploration.

## Data Availability

The datasets used and analysed during the current study are available from the corresponding author on reasonable request.

## Abbreviations

MDT: multidisciplinary team
CBL: case-based learning
LBL: lecture-based learning
